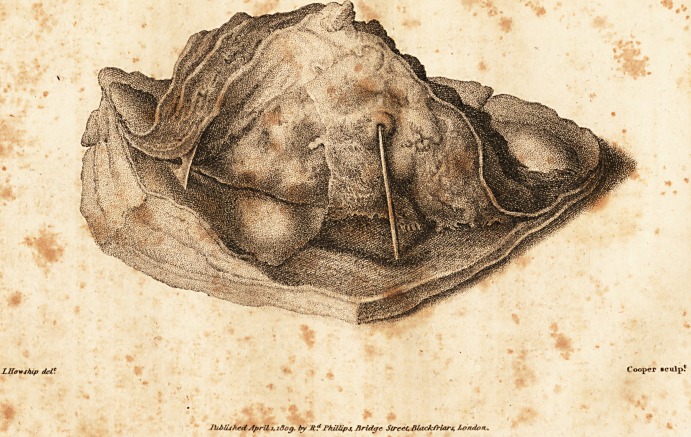# Case of Local Inflammation and Abscess Connected with the Pericardium

**Published:** 1809-04

**Authors:** T. Howship

**Affiliations:** Assistant Surgeon of the 82d Regiment


THE
### Medical and Phyfical Journal.

VOL. XXI.] APRIL, 1809. [NO. 122.

*Printed for R. PHILLIPS, by W. Thorne, Red Lion Court, Fleet Street, London.*

V

### *Case of local Inflammation and Abscess connected with the Pericardium.*

By T. HOWSHIP, Assistant Surgeon of the 82d Regiment.

( With an Engraving. )

A Strong robust man, 48 years of age, from accidental exposure to the night air, took cold. He said he had taken a violent cold. But what is most remarkable is the circumstance of his having at this time repeatedly said to his companions, that he should never recover, but that the cold which he had taken would be his death; while he found so little inconvenience arise, that he still continued to go on with his duty, without making any complaint whatever of sickness to the medical officers of the regiment.

A fortnight after this he was suddenly attacked, after spending the evening with his companions and drinking nearly two pints of ale. He complained of pain in all his limbs, as well as in his head. The pulse was quickened, and the respiration was oppressed. A large blister was immediately laid upon the breast; an emetic was ordered, after the operation of which his feet were immersed in warm water.

The next morning he said he had sweated much; but there was still so great a degree of oppression about the chest, that he was bled to the extent of ℥ xx. This produced almost immediate relief. He once mentioned having a stitch of sharp pain in his breast when he coughed, but he did not feel any thing of this sort after having lost blood.

With a view to relax the surface, he was ordered the saline mixture, which he took frequently during the day, with a low diluent regimen. He had some cough, with which the expectoration was not very free; but fits of coughing, occasionally heavy, were unattended with any pain in the breast.

For three days he continued to take the same medicines, and had a second blister applied, without any decided alteration. His pulse had been so low since the first bleeding, and was so soft and tranquil, beating about 86, with a tongue always clean and moist, that although the skin was dry, it was not considered that his situation would warrant a second bleeding.

On the fourth day he complained of flying pains about the bowels, but not in his breast. As he had been twenty-four hours without a motion, an ounce of Castor oil was given, and he was soon relieved.

On the fifth and sixth days his visage sunk, and his countenance became evidently and greatly altered for the worse. He was free from any sense of pain, and was now not only allowed to take light nourishment in various forms, but was occasionally ordered a little wine also.

On the seventh day of his confinement his respiration gradually became noisy; this change seemed to arise from the difficult transmission of the air through the bronchial ramifications in the lungs. The rattling sound in breathing continued to become more and more aggravated till the evening, in the course of which he expired. To the last half hour of this man's life his intellect remained clear.

During the week spent in the hospital, his answers relating to his sickness were not altogether satisfactory.—From his own account it appeared, that his breast had been quickly relieved by the bleeding, and had since been productive of no uneasiness to him. He had no pain elsewhere. There was, however, a remarkable degree of restlessness and inquietude about him; and this was observed through the night as well as during the day. He would often say he was but very poorly, although he denied that he felt any sort of pain.

Upon examination of the body, the lungs were on the left side, adherent to the pleura lining the ribs and diaphragm as well as the mediastinum, but particularly firm in their connexion with the pericardium.

A section being made through the pericardium and the adherent lung, a small abscess was divided; the contents were of a clear purulent colour, but very consistent. In this part the extent and progress of the disease were manifest. The pericardium was increased to at least four times its natural thickness. The internal surface of this membrane membrane, on the left side, had a bright scarlet aspect, as if very minute effusion had taken place, but nothing like vessels was perceptible. The other parts of the inner surface of the pericardium were of their healthy complexion. Exterior to the left side of the pericardium was an unequally thick stratum of adipose substance; this was deposited between the pericardium and the pleura, external to it. A firm lamina of coagulable lymph had formed the medium of union between this adipose mass and the substance of the lung. The suppurative stage of the disease had formed an irregular cavity in the coagulable lymph, separating the apposed surfaces from each other, and containing nearly half an ounce of fluid matter.

The annexed figure illustrates the diseased appearance of the pericardium, the increased mass of this membrane, and the great proportion of adipose substance on its outside. The portion of lung raised and turned back, shews the appearance of the adherent and the suppurating surfaces.

On one part, the membranes forming the sides of the abscess are seen separated; on another, the lamina of coagulable lymph, being entire, has peeled clean off from the surface of the lung, which surface is seen to possess the characters of health.

Upon inspecting the contents of the abdomen, several spots of bright vascularity were observed upon the peritoneal surface of the mesentary; a clear indication of there having been a temporary increase in the activity of the circulation, which had not however gone on to any length; it probably had been connected with the pains which were felt some days previous to his dissolution, and which were referred to the region of the abdomen and bowels.

The most important observation that arises out of the consideration of this case is perhaps the following, that in this instance there was not any thing like the peculiar kind of sob that was observed to attend the respiration in both the cases (the only cases I have seen) of inflammation of the heart; which circumstance, together with the dissection itself, prove how readily the pericardium may be engaged in inflammation, and consequent disease, without involving either the structure or the actions of the heart.

*Scarborough, March 11, 1809.*

## Figures and Tables

**Figure f1:**